# Insights into replicative senescence of human testicular peritubular cells

**DOI:** 10.1038/s41598-019-51380-w

**Published:** 2019-10-21

**Authors:** Nina Schmid, Florian Flenkenthaler, Jan B. Stöckl, Kim-Gwendolyn Dietrich, Frank M. Köhn, J. Ullrich Schwarzer, Lars Kunz, Manja Luckner, Gerhard Wanner, Georg J. Arnold, Thomas Fröhlich, Artur Mayerhofer

**Affiliations:** 1 LMU München, Biomedical Center (BMC), Anatomy III – Cell Biology, 82152 Planegg-Martinsried, Germany; 2 LMU München, Gene Center, Laboratory for Functional Genome Analysis (LAFUGA), 81377 München, Germany; 3 Andrologicum München, 80331 München, Germany; 4 Andrologie Centrum München, 81241 München, Germany; 5 LMU München, Department Biology II, Division of Neurobiology, 82152 Planegg-Martinsried, Germany; 6 LMU München, Department Biology I, Ultrastructural Research, 82152 Planegg-Martinsried, Germany

**Keywords:** Urinary tract, Ageing

## Abstract

There is evidence for an age-related decline in male reproductive functions, yet how the human testis may age is not understood. Human testicular peritubular cells (HTPCs) transport sperm, contribute to the spermatogonial stem cell (SSC) niche and immune surveillance, and can be isolated and studied *in vitro*. Consequences of replicative senescence of HTPCs were evaluated to gain partial insights into human testicular aging. To this end, early and advanced HTPC passages, in which replicative senescence was indicated by increased cell size, altered nuclear morphology, enhanced β-galactosidase activity, telomere attrition and reduced mitochondrial DNA (mtDNA), were compared. These alterations are typical for senescent cells, in general. To examine HTPC-specific changes, focused ion beam scanning electron microscopy (FIB/SEM) tomography was employed, which revealed a reduced mitochondrial network and an increased lysosome population. The results coincide with the data of a parallel proteomic analysis and indicate deranged proteostasis. The mRNA levels of typical contractility markers and growth factors, important for the SSC niche, were not significantly altered. A secretome analysis identified, however, elevated levels of macrophage migration inhibitory factor (MIF) and dipeptidyl peptidase 4 (DPP4), which may play a role in spermatogenesis. Testicular DPP4 may further represent a possible drug target.

## Introduction

Aging of any given organ is characterized by a decline of its function. This may apply also for the male gonad. It was postulated that with increasing age, an overall decline of male reproductive functions occurs, which involves reduced sperm counts and androgen production, paralleled by structural changes^1,2^. Several investigations indicate that structural and functional changes in the testis of men in advanced age involve the androgen-producing Leydig cells and the Sertoli cells, which are regarded as the nurse cells for male gem cells^3–7^. In addition, age-related alterations of the tubular wall compartment (also called *tunica propria*) in men were reported. These changes include progressive enlargement and sclerosis, accompanied by impaired spermatogenesis and (in some cases) complete tubular sclerosis^2,5,6^. In contrast, a study in men with proven fertility, who were examined later in life, suggested that aging of the human testis may not necessarily be associated with structural/functional changes, in general^8^. Hence, there is debate about the selection criteria of men in the mentioned studies, as patients with pre-existing infertility and accompanying structural changes may have been included^2,5,6^. In addition, all these investigations in elderly men have limitations with respect to statistically relevant amounts of samples. Conceivably, age-related alterations of testicular structure and function in men are likely a consequence of many factors acting together in the long-lived human species. Besides age itself, medical conditions, use of drugs and overall lifestyle can affect both, the regulation of testicular functions and/or the male gonads itself. These issues cannot be well separated in human and there is no established, long-lived animal model to adequately mirror this situation.

A possible approach to investigate testicular aging would be the examination of senescence of testicular cells, yet most human testicular cells cannot be propagated *in vitro*, with the exception of peritubular cells. Human testicular peritubular cells form several layers within the wall surrounding the germinal epithelium and morphological studies suggested that they may change during testicular aging^2,5,6^. Peritubular cells of the adult testis are contractile, smooth muscle-like cells and secrete extracellular matrix (ECM) proteins. Imbalances between secretion/maintenance of the ECM and cellular contractile abilities are documented in the testes of men with impaired spermatogenesis^9,10^. The resulting fibrosis of the tubular wall is furthermore accompanied by accumulation of immune cells, implying a sterile type of inflammation in the tubular wall of infertile men^10–12^. As mentioned, human testicular peritubular cells (HTPCs) can be isolated and cultured from human testicular tissue^13^. HTPCs produce important growth factor molecules, e.g. glial cell line derived factor (GDNF) and C-X-C motif chemokine ligand 12 (CXCL12) and thereby most likely contribute to the spermatogonial stem cell (SSC) niche of the testis and to life-long spermatogenesis^14–16^. Initially, Sertoli cells were known as sole producers of GDNF and an overall important part of the SSC niche. Yet the importance of complementary GDNF, derived from peritubular cells, was confirmed in a systemic animal model^17^. The GDNF-mutant mice lost their fertility with increasing age. This observation links aging to a function of peritubular cells and concurs with the concept that impairments of the SSC niche may be part of testicular aging^18^. In this context a recent study of the human testes by Pohl *et al*.^19^ documented the occurrence of diminished spermatogenic efficiency, increased amount of proliferating A_dark_ spermatogonia and altered nuclear morphology of Sertoli cells with increasing age. This suggests alterations of Sertoli cell function. It does not rule out changes in peritubular cell functions, although obvious structural alterations of the tubular wall or peritubular cells with increasing age in men were not described.

HTPCs also secrete cytokines, express Toll-like receptors, purinergic receptors and produce reactive oxygen species (ROS)^20^. Previous work implies that peritubular cells are involved in sterile inflammation, associated with some cases of male infertility^10,21,22^. Inflammation may also be part of aging. Indeed the term “inflammaging”^23^ was coined. A possible functional involvement of peritubular cells in aging of the human testis was not examined yet. We reasoned that replicative senescence, triggered by serial passaging of cultured HTPCs, may allow the investigation of their contribution to testicular aging. Replicative senescence is an established cell culture model for research in aging and senescence^24,25^. It describes a cellular response, which limits proliferation of aged or damaged cells, in association with restricted cellular function^26^ and a characteristic secretory phenotype, i.e. senescence-associated secretory phenotype (SASP)^27^. The consequences of replicative senescence in early and advanced passages of cultured HTPCs were investigated employing complementary approaches, including proteomics, qPCR studies and light microscopy (LM) combined with focused ion beam scanning electron microscopy (FIB/SEM) tomography, which allowed 3D reconstructions of various cellular compartments.

## Results

### Replicative senescence in HTPCs

**Figure 1:**
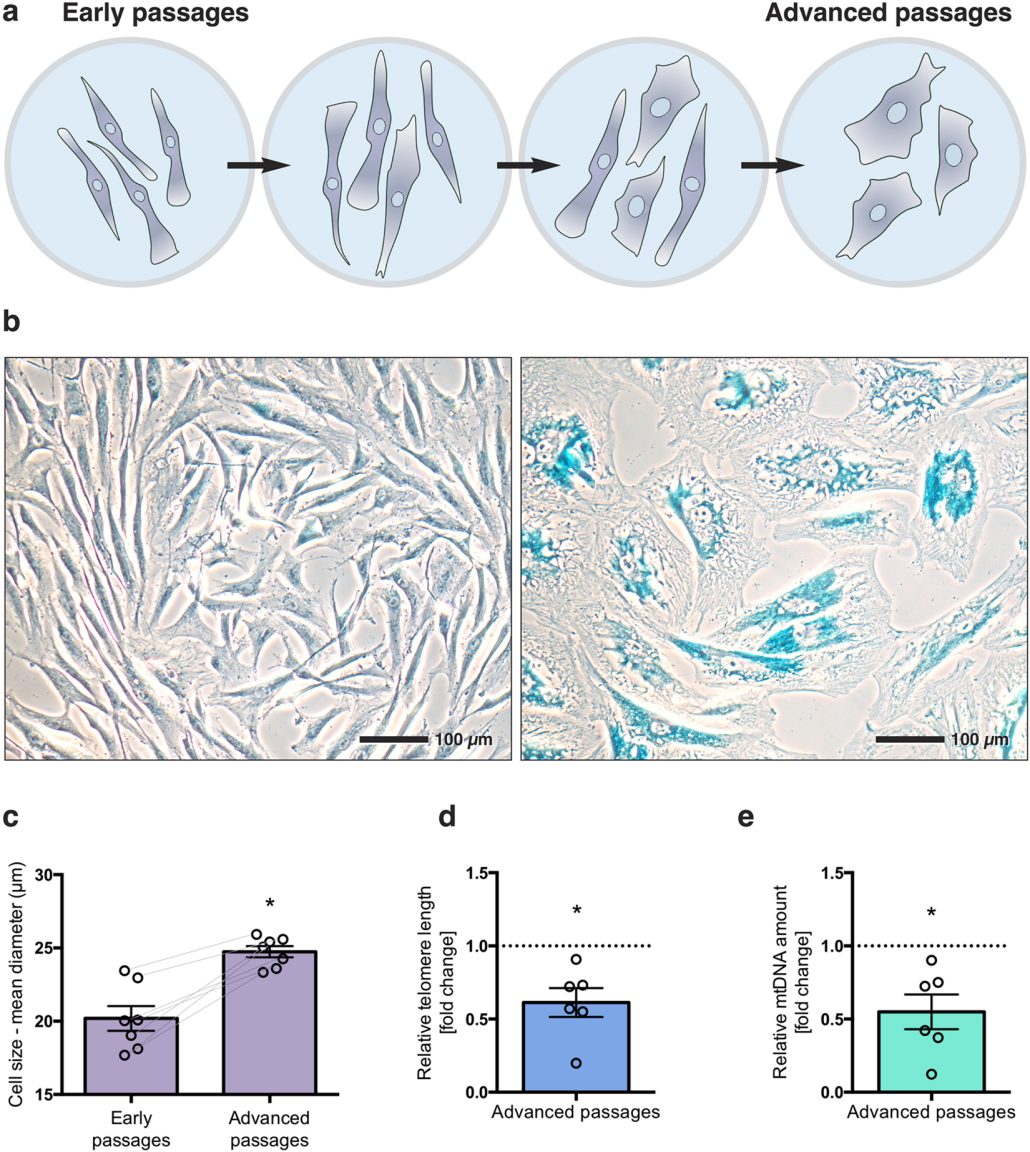
Changes associated with replicative senescence in HTPCs. Schematic presentation of HTPCs in transition from early to advanced passages (**a**). Light micrographs of senescence associated β-galactosidase staining of HTPCs: in contrast to HTPCs from early passage (passage = P; P5, left), β-galactosidase activity is prominent in advanced passage (P15, right) (**b**). Measurement of cell sizes revealed a significant increase in diameter in advanced passages from the same donor (n = 7), statistics were calculated with a paired two-sample *t*-test (**c**). Quantification of the relative telomere length from isolated gDNA from advanced passages of HTPCs from the same donor (n = 5) with significant telomere attrition, normalized to early passages (**d**). Quantification of mtDNA copy number by qPCR indicates significantly decreased amount of mtDNA in advanced HTPC passages (**e**). Statistical analysis was done with an one-sample *t*-test (**d**,**e**), mean ± SEM are given, asterisks indicate statistical significance, *p < 0.05, **p < 0.01, ***p < 0.001.

HTPCs divide in culture with a doubling time of 2–3 days and can be propagated until cell division ceases after approximately one year. Cells from the same donors without (low passage number) and with signs of replicative senescence (advanced passage number) were compared. In addition to growth arrest, the following criteria were used to define replicative senescence of HTPCs: a change of the spindle-like morphology to an irregularly shaped and flattened morphology (Fig. 1a), increased β-galactosidase activity (Fig. 1b), increased cell size (Fig. 1c) and reduced telomere length (Fig. 1d). Cells fulfulling these criteria were further examined. Furthermore, we found that mtDNA was reduced in advanced passges, compared to the corresponding early passages (Fig. 1e).

### FIB/SEM tomography of early vs. advanced passages of HTPCs

**Figure 2:**
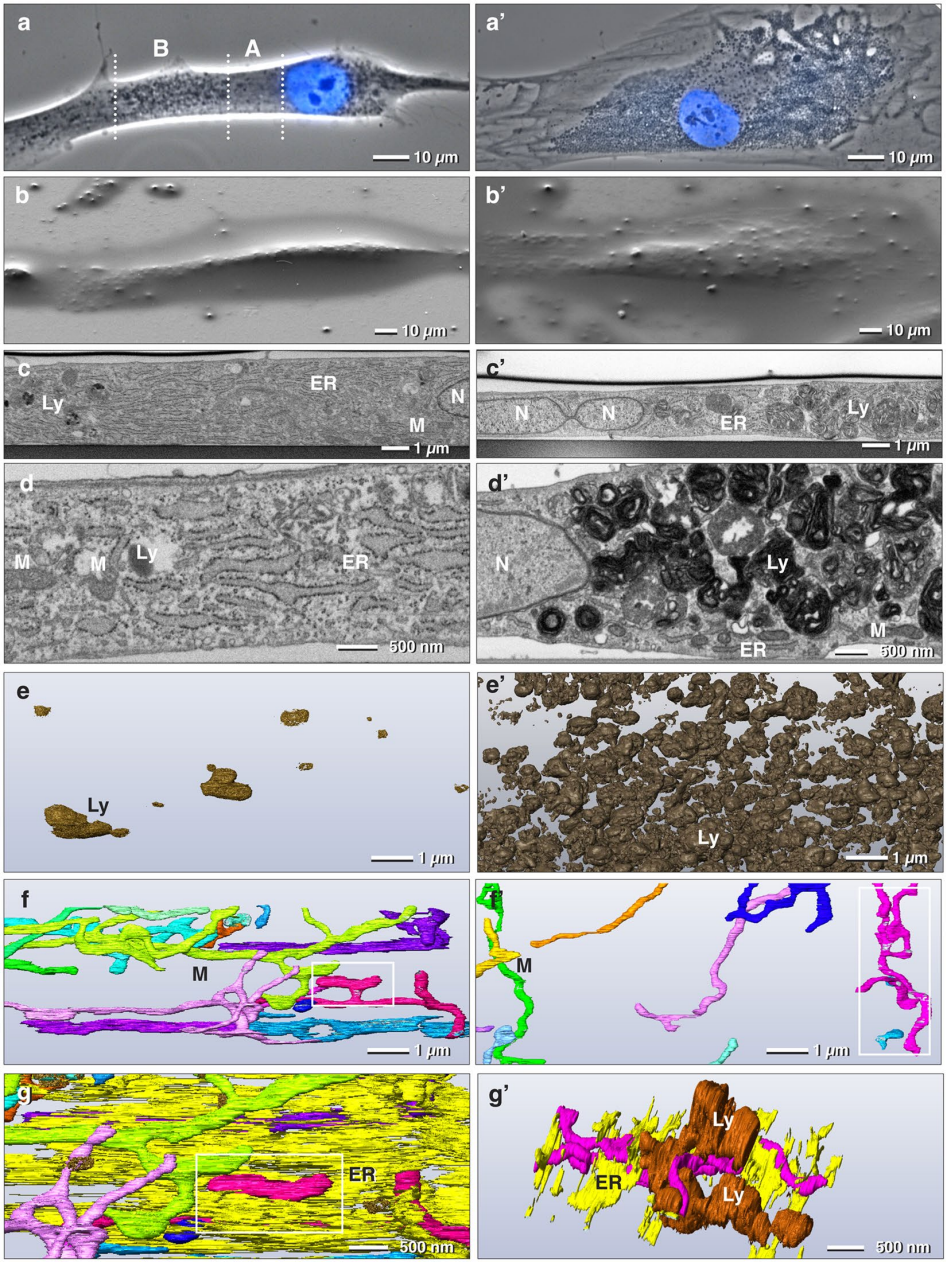
Focused ion beam scanning electron microscopy (FIB/SEM) tomography of early *vs*. advanced passages of HTPCs. Correlative LM and FIB/SEM tomography of ultrastructural changes of HTPCs (early passages: **a**–**g**; advanced passages: **a’**-**g’**). In early passages the cells are spindle-shaped with a rather homogeneous region of cytoplasm proximal to the nucleus (**a**: A) and a distal, granular region (**a**: B). Senescent cells flatten out and their cytoplasm becomes packed with granules (**a’**). Selected cells from LM were re-localized with SEM (**b** and **b’**) and sectioned with FIB longitudinally (**c**,**c’**; **d**,**d’**). In early passages the homogeneous part of the cytoplasm is characterized by large, parallel sheets of rough endoplasmic reticulum (ER) and granules, which are classified as lysosomes (Ly) (**c–e**), moderate electron dense after osmium fixation (**c**,**c’**) and very electron dense after rOTO-staining (**d**,**d’**). In senescent cells lysosomes accumulate in enormous numbers (**c’**,**d’**,**e’**). Mitochondria are elongated and form branched larger networks, independent of the passage (**f**,**f’**; individual mitochondria are differently colored). However, in senescent cells the portion of mitochondria related to the cytoplasm is significantly reduced (**f**,**f’**). The ER is densely packed with ribosomes during all stages and is associated with both, mitochondria (**g**,**g’**) and lysosomes (**g’**). M = mitochondrion; N = nucleus

Ultrathin embedding of HTPCs allowed identification of target cells in SEM due to the coordinate system engraved on the slides and the specific cell topography. Superposition of LM (phase contrast) and SEM images allowed rapid correlation of fine structural details in the nanometer range. HTPCs of early passages are elongate, spindle-shaped cells, with a prominent nucleus and several nucleoli (Figs 1a, 2a,b). In phase contrast, granular substructures dispersed within the cytoplasm were observed in all cells. At one side of the nucleus the cytoplasm appeared to be devoid of larger substructures over a distance of 10–20 µm (Fig. 2a). In contrast, cells of advanced passages exhibited an abounding number of granules, either bright or dark in phase contrast, evenly distributed within the cytoplasm (Fig. 2a’). For better insight into the distribution of cellular substructures in relation to the nucleus, longitudinal sections were chosen for FIB/SEM analysis of both passages (Fig. 2c,c’,d,d’).

Cells in early passages were rather flat with a height in the range of 3 µm (Fig. 2c). The nucleus was lens shaped with parallel envelope membranes. Nuclear pores could be visualized by volume rendering of the 3D-data set. Several strands of ER were typically connected to the outer nuclear membrane. The ER formed: i) large, parallel sheets densely packed with ribosomes, ii) smaller sheets of fenestrated rough ER, randomly orientated and iii) bulb shaped dilatations with either electron translucent or fine granular lumen (Fig. 2c,d). Mitochondria were up to 15 µm long and very variable in their diameter, ranging from 0.3 µm to 0.1 µm, without any cristae. Only in the 3D context could they be recognized as mitochondria (Fig. 2c,d,f). Most mitochondria were branched and formed a complex network including numerous loops. Characteristic for all mitochondrial networks were multiple intimate contact sites with sheets of rough ER (Fig. 2g). The mitochondrial tubes were locally tightly enwrapped so that the membranes of ER and the outer mitochondrial membrane could not be discriminated, even at the resolution limit of FIB/SEM (2 nm isovoxel). At the site of contact, the ER was depleted of ribosomes. There was a population of roughly globular organelles composed of two components: a very electron dense matrix of packed/and or fused membranes and electron translucent areas resembling vacuoles, ranging from approximately 200 nm to 2 µm in diameter (Fig. 2c). When present in higher densities, they were interconnected. They were regarded as lysosomes, rather than autophagosomes or autophagolysosomes, as there was no indication for the presence of membranous phagophores enwrapping cell organelles^28^, e.g. mitochondria. When examining lysosomes in a 3D context, it became evident that the ER lumen was in continuity to both, the vacuolar, and the membrane part of the lysosomes. In early passages lysosomes were not distributed randomly within the cell but rather accumulated in one half of the cell, typically 10–15 µm distal from the nucleus. This concurs with the granular structures observed in the phase contrast images (Fig. 2a, a’).

**Figure 3:**
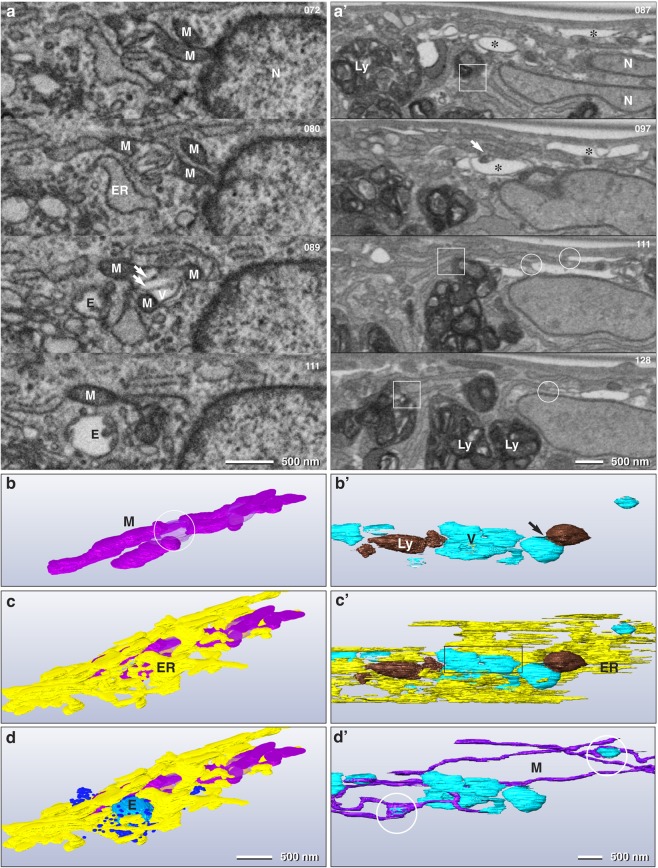
Selected FIB/SEM micrographs of a series of HTPCs from early (**a**–**d**) and advanced passages (**a’**–**d’**). The numbers (upper right) indicate the selected micrographs from the series. In the early passage the HTPC shows contact sites of ER with a branched mitochondrion (M) with several cross sections within one micrograph (4 nm isovoxel). Vacuoles (V) form within the mitochondrial matrix, leading to swellings (**b**; circle/transparent magenta) with some cristae still visible (**a**, arrows). Sheets of ER are attached to the mitochondrial network, noticeable after 3D reconstruction (**c**,**d**). The advanced passage exhibits numerous lens shaped vacuoles (**a’**; asterisks; voxel size: 7.5 × 7.5 × 14 nm). Strands of rough ER are connected at both ends to the vacuoles (**a’**, circles). Electron dense inclusions within the vacuole were identified as lysosomes (**a’**, arrow), when following the FIB/SEM series. Lysosomes (Ly) are connected to strands of ER at multiple sites (**a’**; squares). 3Dreconstruction of vacuoles (V) and lysosomes (Ly) show that they form clusters connected to each other (**b’**; arrow). The ER forms large sheets, best visible in top view (**c’**) compared to the FIB/SEM block face micrographs (**a’**). The ER membrane is in continuity with the vacuole membrane (**c’**; rectangle). The mitochondrion (M) shown is also locally fused with smaller and larger vacuoles (**d’**; circles).

HTPCs in advanced passages lost their original spindle-like shape and flatten out to irregular shapes (Fig. 2a’, b’, c’). Although the nucleus still appeared to be roughly lens shaped in LM, deduced from DAPI images (Fig. 2a’), the 3D analysis revealed numerous cytoplasmic invaginations (Fig. 2c’). Nuclear pores were still visible with volume rendering of the 3D data set. The mitochondrial networks were pertained (Fig. 2f’), however, their number decreased in relation to the corresponding cytoplasmic volume (normalized to 100 µm^3^) (Table 1). Deducing from statistical measurements, the mitochondria (network) became smaller in diameter (0.22 µm *vs*. 0.18 µm) but extended in length (5.8 µm *vs*. 8.8 µm) (Fig. 2f’). However, the total mitochondrial surface in a given volume of 100 µm^3^ was reduced to approximately 25% compared to cells of early passages. The characteristic contact sites between mitochondria and ER were preserved, however, the shape of ER changed from large parallel sheets to smaller slabs with random orientations (Fig. 2g’). There was indication for at least partial degradation of mitochondria or mitochondrial segments, which became visible upon large volume serial block face sectioning: the mitochondrial matrix became locally electron translucent, formed blebs, with electron dense membrane fragments, likely residual cristae (Fig. 3a,b). The 3D reconstruction revealed that several segments of the mitochondrial network can be interconnected by vacuolated blebs (Fig. 3d’; Supplementary Fig. 1). Endosomes and small vesicular structures were in close vicinity to both, ER and mitochondria (Fig. 3a,c,d). Within the sheets of rough ER, small lens-shaped vacuoles were frequently observed (Fig. 3a’) and several vacuoles of different sizes can be interconnected *via* rough ER (Fig. 3c’). The most striking structural alteration was an enormous accumulation of lysosomes (Fig. 2d,d’; e,e’, 3a’). Their volume portion increased almost tenfold (from 7% to 60%). 3D reconstructions revealed that most lysosomes, are interconnected, either directly by their electron dense and electron translucent components or by short strands of ER (Fig. 2e’, 3a’). The ER was in luminal connection to the lysosomes (Fig. 3a’; Supplementary Fig. 1). The intimate contact of the mitochondria with sheets of rough ER was pertained, as shown by 3D reconstructions (Fig. 3c,c’, d).

**Table 1:** Morphological changes of cell organelles.

	Early passages	Advanced passages
Number of mitochondria/100 µm^3^ *	20	6
Mean volume of mitochondrion	0.22 µm^3^	0.22 µm^3^
Mean surface area of mitochondrion	7.5 µm^2^	6.5 µm^2^
Average diameter of mitochondrion	0.22 µm	0.18 µm
Calculated length of mitochondrion (L = V/A) V = Volume, A = Area	5.8 µm	8.8 µm
Volume portion of lysosomes*	7%	60%
Volume portion of ER*	31%	4%

^*^Related to cytoplasmic volume.

### Proteomic approach for comparison of early vs. advanced HTPC passages

**Figure 4:**
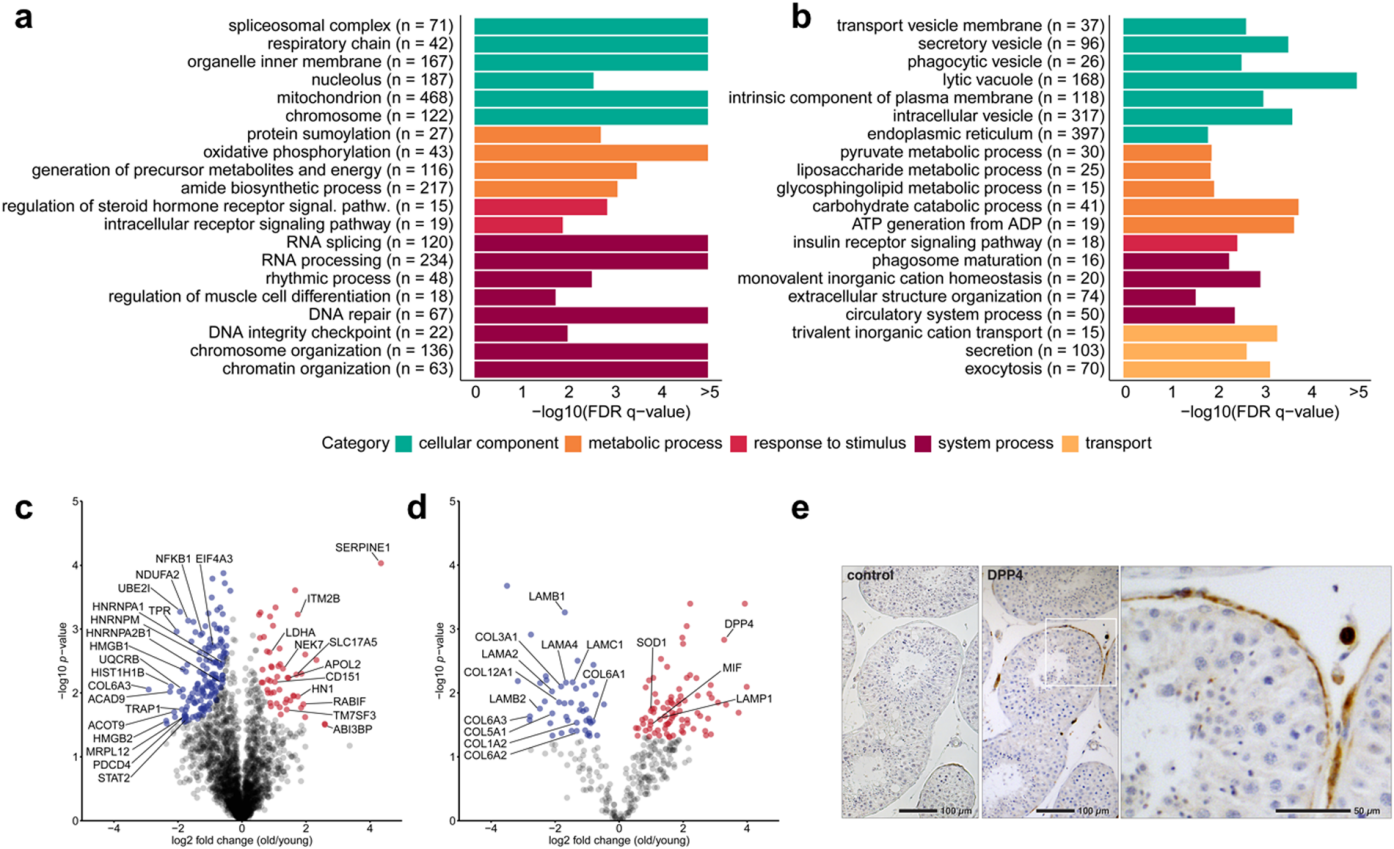
Proteomic analysis of advanced compared to early passages of HTPCs. Gene set enrichment analysis (GSEA) revealed significantly enriched gene sets (FDR q-value ≤ 0.05) and were summarized using REVIGO by clustering semantically similar GO terms. Each of the 20 characteristic gene sets enriched in early (**a**) and advanced passages (**b**) of HTPCs are shown. Color-coding refers to the corresponding highest GO hierarchy level. The x-axis shows the enrichment significance resulting from the GSEA and is depicted as –log10 (FDR q-value). The number of quantified proteins per gene set is shown in brackets. Volcano plots of intracellular and extracellular proteins, which are more abundant in passaged HTPC cellular proteomes (**c**) and secretomes (**d**) are depicted as red dots and proteins less abundant are shown as blue dots, respectively. Selected proteins with significant difference in abundance are labeled. P-values were calculated by a paired two-sample *t*-test. DPP4 expression in testicular peritubular cells (**e**). Light micrographs of immunohistochemical staining of human testicular sections. DPP4 is detected in several peritubular cells and cells of the interstitial space. Right micrograph: detail of the DPP4 staining (framed area). The negative control is without staining.

A quantitative proteome analysis of early and advanced passages of cultured HTPCs (n = 6) and their conditioned media (CM) was performed to explore potential molecular alterations of replicative senescence of the HTPC proteome and secretome. A total of 3692 proteins were identified in HTPC-lysates at a false discovery rate (FDR) < 0.01. All identified proteins in early and advanced HTPC passages are listed in the Supplementary Table 1. Proteome analysis of CM identified 860 proteins, including 569 proteins with annotated extracellular localization, defining the secretome dataset. The identified proteins in CM of early and advanced HTPC passages are listed in the Supplementary Table 2. Hierarchical clustering and principal component analyses demonstrated passage-associated segregation of cellular proteomes and secretomes from early and advanced passages (Supplementary Fig. 2a,b). Label-free quantification (LFQ) revealed that 208 proteins were significantly different in quantity (*t*-test, FDR < 0.05) in cellular proteomes **(**Fig. 4c, Supplementary Table 3**)** and 131 proteins in the secretomes **(**Fig. 4d, Supplementary Table 3**)**, between HTPCs from early and advanced passages, respectively. A large number of nuclear and mitochondrial proteins were decreased in contrast to increasing lysosomal proteins (Fig. 4a,b).

The secretome analysis of advanced passages showed, among others, a significant increase of the dipeptidyl peptidase 4 (DPP4) (Fig. 4d). As DPP4 expression has not been identified before in testicular peritubular cells, we explored its presence *in situ*. DPP4 staining was carried out with human testicular sections from middle-aged men (48–50 years). DPP4 was absent in many of the peritubular cells of the seminiferous tubular wall. However, in half of the samples examined (3 out of 6 examined) immuno-reactive peritubular cells were found *in situ*. DPP4 was also found in cells of the interstitial space (Fig. 4e). The gene set enrichment analysis (GSEA) showed substantial alterations between early and advanced passages in cell lysates and CM. The gene sets enriched in early passages were primarily related to mitochondrial and chromosomal functions, and more specifically related to RNA splicing, chromatin organization and DNA repair (Fig. 4a), while secretory, lysosomal, and metabolic processes were enriched in advanced passages (Fig. 4b). In the HTPC CM, gene sets related to extracellular matrix were enriched in early passages, while gene sets related to different binding and signaling processes were enriched in advanced passages. The detailed results of the GSEA are listed in Supplementary Tables 5, 6 and are visualized as functional networks in Supplementary Fig. 3.

### qPCR study of characteristic HTPC marker genes

**Figure 5:**
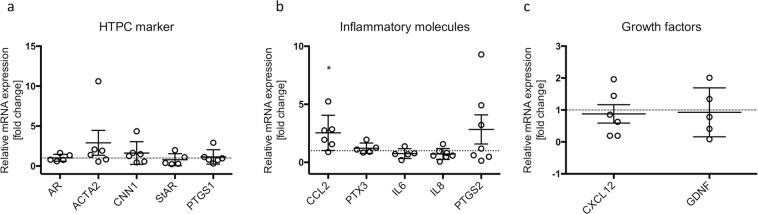
qPCR study of typical genes expressed in peritubular cells. mRNA levels of characteristic HTPC marker genes like *AR*, *ACTA2*, *CNN1*, *StAR* and *PTGS1* (**a**). Inflammation-associated genes show significantly increased mRNA level of *CCL2*. *PTX3*, *IL6*, *IL8* and *PTGS2* are not changed (**b**). mRNA expression of growth factors, *CXCL12* and *GDNF* (**c**). Graphs represent individual measurements and means ± SEM. Statistical analysis was executed with one-sample *t*-test, Asterisks show statistical significance, *p < 0.05, **p < 0.01, ***p < 0.001.

For complementation of the study, possible alterations between early and advanced passages were examined by qPCR. Transcription levels of *AR*, *ACTA2*, *CNN1*, *StAR* and *PTGS1* remained stable (Fig. 5a). Inflammatory genes, namely *IL6*, *IL8*, *PTGS2* and *PTX3* were also not affected by passaging except for *CCL2*, which was increased about 2-fold (Fig. 5b**)**. However, ELISA-measurements did not confirm such a change at the protein level (Supplementary Fig. 5). Expression levels of the growth factors *CXCL12* and *GDNF* varied between the different donors, although without detectable tendency (Fig. 5c). ELISA-measurements of CXCL12 did not indicate a significant age-associated change, but rather inter-individual alterations (Supplementary Fig. 4**)**.

## Discussion

In man, aging of many organs, including the testis, cannot be readily assessed. Information about cellular senescence of human testicular cells is likewise missing. HTPCs are the only human testicular cell type, which can be cultured and propagated until replicative senescence is witnessed. The present study combines ultrastructural 3D data from HTPCs during senescence with a proteomic analysis of cellular and secreted proteins and a qPCR analysis and provides a detailed picture of senescence-associated changes in these human testicular cells.

In general, replicative senescence of cells is characterized by cell cycle arrest^29,30^, changes in the cellular phenotype and telomere shortening^31^. As there is no universal marker, the combination of different hallmarks is typically used to characterize senescent cells^32^. All HTPCs analyzed in this study showed a combination of impaired proliferative competence, reduced telomere length, increased cell size, and increased β-galactosidase activity, i.e. the general hallmarks of senescent cells^29,31,33,34^. The study of these cells allowed us to identify HTPC-specific changes, which may be of relevance for the functions of the testis.

Detailed insights into cellular compartments of HTPCs, based on 3D reconstruction, identified three significant ultrastructural changes, associated with cellular senescence in HTPCs: i) a significant decrease of rough ER ii) a dramatic increase of lysosomes, iii) a decrease in the number of mitochondria. Ultrastructural reconstructions revealed that the ER is central in all these changes. It is connected to all lysosomes (Fig. 3a’), is in intimate contact to all mitochondria (Figs. 2g,g’: 3c), and involved in vacuole formation (Fig. 3a’). The ER was packed with ribosomes in a surprisingly high density in early and advanced passages, hence an efficient protein synthesis is visibly maintained in senescent cells. Both *in vivo* and *in vitro* (human) testicular peritubular cells secrete a plethora of proteins, mainly ECM components^15^. The proteomic data supported the general capacity for protein synthesis during all passages, however secreted ECM proteins are significantly decreased, concurring with the structural reduction of the ER from 31% to 4% of the cytoplasmic volume (Table 1). These results are in line with impaired protein homeostasis (proteostasis) in senescent HTPCs, which is associated with aging in many cells^35^.

The striking increase of lysosomes, which make up 60% of the cell volume in advanced passages, further argues for impaired proteostasis as a central event. The 3D reconstructions showed that in HTPCs lysosomes were connected to the ER in early and advanced passages (Fig. 3a’; Supplementary Fig. 1). Only in early passages, cellular polarity was observed with respect to a region located at one side of the nucleus, which was almost free of lysosomes and occupied by accumulation of parallel-arranged large sheets of rough ER (Fig. 2c,d). This cellular polarity was lost gradually in advanced passages. The massive accumulation of lysosomes reduced the space available for rough ER and implies steric hindrance of formation of rough ER. Similar data were recently published for large volume FIB/SEM reconstructions of HeLa cells: the dictyosomes, endosomes, lipid bodies and lysosomes form an interconnected system for Golgi degradation and reconstitution^36^. The massive increase of lysosomes, both in number and volume, may have different reasons. Thus, together with the proteomic data (Fig. 4b) and published physiological *in vivo* data^37^ the results indicate impaired proteostasis.

Small vacuoles are initially visible within rough ER sheets as lens shaped structures (Fig. 3a’) and subsequently, larger, spherical structures, still in luminal contact with ER, were found. They were considered to be nascent lysosomes and it seems likely that the formation of the vacuolar part of the mature lysosomes is a consequence of direct involvement of ER membranes and ER lumen. Similar autophagolysosomes/autophagosomes, degrading mitochondria, are described in podocytes of rats after acute ischemia^38^ and in hexa KO cells, shown in serial 3D reconstruction, and also indicateinvolvement of ER^39^.

**Figure 6:**
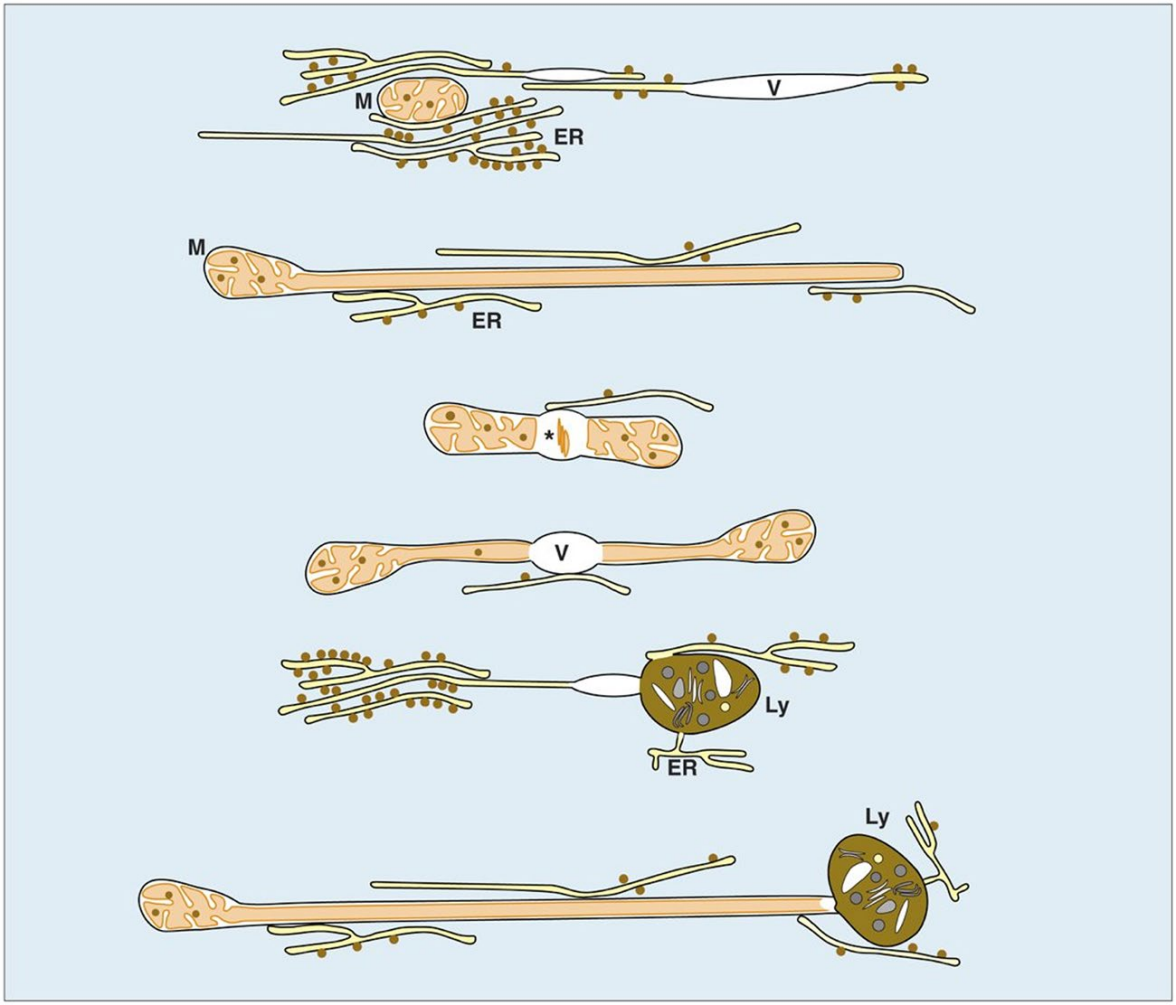
Schematic representation of the network of endoplasmic reticulum (ER), mitochondria (M), lysosomes (Ly), vacuoles (V) and their mutual relations based on electron microscopy data. All partners can be connected *via* ER. Sheets of rough ER enwrap mitochondria. Small lens shaped vacuoles form within the ER lumen. Mitochondria are elongated and range up to 15 µm. They have a minimal diameter of approx. 100 nm, without any cristae present. With progressing senescence, vacuoles form within the mitochondrial matrix, sometimes with degenerating cristae visible (asterisk). Both, mitochondria and rough ER are in direct contact to lysosomes forming a common, interconnected system.

The contact sites of ER with mitochondria are being discussed for Ca^2+^ exchange^40^ but also as a supply site of membrane components from the ER to the outer mitochondrial membrane^41^. Changes of the mitochondrial network and the reduction in surface area of mitochondria by a factor of four was qualitatively paralleled with a reduction of the rough ER (Table 1). The investigation of lysosomes revealed that the majority is composed of an electron dense matrix, which is, at least in part, formed by an aggregation of membranes. However, when looking at the mitochondria with large volume reconstruction, there are characteristic features: empty spaces, lacking cristae, within the mitochondrial matrix, similar in appearance to data from Szento *et al*.^38^ and contacts of mitochondria with lysosomes (Fig. 2g’; Supplementary Fig. 1) even at several segments of individual mitochondria, which may explain the reduction of mitochondrial volume by degradation (Fig. 3a**;** Supplementary Fig. 1). Compared to typical autophagy of cell organelles, as reviewed by Feng *et al*.^42^, where entire mitochondria or other cell organelles are engulfed by membranes to form an autophagosome^43^, the observed mechanism in HTPCs seems to be different. The mitochondrial vacuoles may enable fusion with ER-derived vacuoles and/or existing lysosomes. Mitochondria and rough ER are in direct contact to each other, as well as to lysosomes, thus forming a common, interconnected system shifting with progressing senescence to lysosome formation (Fig. 6).

Mass spectrometry revealed further striking changes of the cellular protein pattern, including the ubiquitous transcription factor NFκB1 that showed lower levels in senescent HTPCs. It was reported that a loss of NFκB1 may lead to early onset aging^44^. Furthermore, a reduced abundance of the RNA binding protein HNRNPA1 was found. It controls cellular senescence and the SASP *via* sirtuin1. Loss of HNRNPA1 induces a senescent phenotype in human diploid fibroblasts^45^ also due to its crucial role in telomere protection^46^. With increasing age, energy production is reduced as reviewed by Barzilai, *et al*.^47^. The GSEA results of the proteome suggest that the energy metabolism changes in senescent cells from an oxidative metabolism to anaerobic glycolysis, which correlates with decreased mtDNA, structural changes within the mitochondria as reviewed by Bratic *et al*.^48^ and increased LDHA abundance in advanced HTPC passages^49^. Senescent cells show changes in chromosome and chromatin architecture in general, as reviewed by Sun *et al*.^50^. GSEA showed that chromosome and chromatin organization are changed in advanced passages (Fig. 4a,b). This is paralleled by alterations of the shape of the nucleus, seen in many HTPCs (Fig. 3a’). In addition, the reduced DNA repair capacity in advanced passages, in combination with age-related increased levels of DNA damage, could reinforce cellular senescence^51^.

ROS production by HTPCs was described in previous studies^20^. The analysis of the secretome revealed that a number of factors, e.g. superoxide-dismutase 1 (SOD1), an antioxidative factor, were elevated in advanced passages. This may be a reaction to higher ROS levels, which can cause DNA damage, leading to cellular senescence in human fibroblast cell lines^52^. MIF, a pro-inflammatory cytokine, increased in senescent HTPCs, is expressed by immune cells and several other cell types, including smooth muscle cells^53^ (Fig. 4d). MIF is released into the extracellular space in response to various stimuli (e.g. mitogens and pro-inflammatory cytokines), and elevated levels indicate a pro-inflammatory milieu. MIF binds to cell surface receptors, either CXC chemokine receptors (CXCRs) or the CD74 receptor^54^. The G-protein–coupled chemokine receptors CXCR2, −4, and −7 are linked to chemotaxis of immune cells, in general. MIF could thereby contribute to an inflammatory environment within the human testis, also *via* suppression of the anti-inflammatory effect of glucocorticoids^55^. MIF actions may lead to enriched cytokine levels and can activate important pathways in senescence and aging, namely p53 and NFκB as reviewed by Salminen *et al*.^56^.

Within the testis, a special situation may exist, since MIF is a ligand for the CXCR4, expressed by SSCs, and is essential for postnatal maintenance of the SSCs in marmosets and mice^57^. If indeed applicable to the testis, higher levels of MIF may influence the SSC niche *via* CXCR4 binding. CXCR4-expressing cells in the testis may also be indirectly influenced by DPP4, which was recently described as a senescence marker for human fibroblasts^58^. DPP4 was significantly elevated in the secretome of advanced HTPC passages (Fig. 4d) and detected *in vivo* in human testicular samples in peritubular cells (Fig. 4e). DPP4 is able to cleave several neuropeptides but also chemokines, including CXCL12. This is of importance in hematopoiesis, angiogenesis, and stem cell homing. With respect to the testis, it was shown that at least in mice and marmosets CXCL12-CXCR4 signaling is required for postnatal maintenance of SSCs, SSC propagation and that it prevents SSC differentiation. As CXCL12 truncation by DPP4 can reduce CXCL12-CXCR4 signaling significantly^59^, DPP4 within the testis may have a negative impact on spermatogenesis and may antagonize other chemokine- and neuropeptide actions. The expression of DPP4 in the human testis, not shown before, raises the possibility that inhibitors for DPP4 may be potentially useful for therapy of diseases of the testis or age-related changes. In this respect, gliptins, i.e. established anti-diabetic drugs^60^ may be of interest, as they are inhibitors of DPP4 and were shown to affect stem cell niches^61^.

The levels of several ECM factors, including various laminins and collagens, are reduced in the secretome of advanced passages (Fig. 4d). They represent the major fractions of secreted proteins of HTPCs^15^, and reduced levels indicate an overall diminished secretory activity and/or impaired proteostasis of senescent HTPCs. Yet a reduced secretion is not in line with reported increased ECM-deposits in the tubular wall of elderly men^6^ or the changes seen in old rodent testes^62^. The human data is, however, being debated and questioned. It is possible that men with pre-existing infertility may have been included. As fibrosis of the peritubular wall is a hallmark of male infertility, such an inclusion may conceivably have biased the conclusion. If the results of the present study correlated with the *in vivo* situation, fibrosis of the peritubular wall is not be expected in testes of healthy elderly men without confounding issues^8^. Indeed, a recent light microscopic study by Pohl *et al*.^19^ did not detect differences in the tubular wall compartment from young and elderly men. Further, peritubular cells are smooth muscle-like cells, comparable with other smooth muscle cells of the body. Of note, senescent vascular smooth muscle cells, like senescent HTPCs, also showed reduced ECM protein secretion^63^.

Several distinct factors of importance for HTPCs (AR and GDNF), were not readily detectable by LC-MS/MS, but could be analyzed by qPCR (Fig. 5). *AR* was not significantly altered in advanced passages. AR-activation enhances the smooth muscle-phenotype and thereby may regulate contractility of HTPCs^16^. In full accordance with the proteomic data, the smooth muscle factors *ACTA2* and *CNN1* also remained stable, thus HTPCs keep their smooth muscle-like phenotype. GDNF contributes to spermatogenesis in mice^14,17^ and was also unchanged in advanced passages, like protein (LC-MS/MS) and mRNA levels of *PTGS1*. This enzyme is involved in the generation of prostaglandins and thereby in the regulation of GDNF^64^. CXCL12 also contributes to spermatogenesis and the mRNA and protein levels (Supplementary Fig. 4) were likewise not significantly altered. For all these transcript levels, considerable variations between individual human samples were, however, observed.

Several pro-inflammatory factors were also examined (*IL6*, *IL8*, *CCL2*, *PTGS2* and *PTX3)*, only *CCL2* expression was slightly, but statistically significantly (about 2-fold) elevated in senescent HTPCs. This cytokine was identified in HTPCs^21,22^ and is involved in attracting monocytes and thus is considered a pro-inflammatory factor^65^. A related smooth muscle cell type in blood vessels showed higher levels of CCL2 with age, causing an inflammatory environment^66^. However, ELISA-measurements showed that not necessarily CCL2 content was changed in the cell culture media of HTPCs (Supplementary Fig. 5).

In summary, the results describe details of cellular senescence in human testicular cells. Senescence in HTPCs is not associated with a decreased expression of crucial HTPC genes, including contractility markers, *AR* and the growth factors *GDNF* and *CXCL12*. However, striking morphological changes in senescent HTPCs are accompanied by altered cellular protein levels, indicating impaired proteostasis. The secretome analysis revealed that specifically ECM-components are reduced. In contrast, inflammatory factors are increased. The HTPC-specific SASP includes MIF and DPP4, which both, directly or indirectly could influence the SSCs. In particular, DPP4 may be involved in degrading CXCL12. Thereby it could negatively influence the SSC niche. If so, it may serve as a potential drug target.

Reliable data on testicular aging and associated functional and structural changes in man are missing to the very day. Hence, the full *in vivo*-relevance of the changes in the proteome and secretome of HTPCs, in combination with ultrastructural large volume 3D-data, for the understanding of testicular aging in man remains to be shown.

## Methods

HTPCs were isolated from testicular tissue fragments as described earlier^13,21^. The study was approved by the local ethical committee (Ethikkommission, Technische Universität München, Fakultät für Medizin, project number 309/14). For the scientific use of the tissue samples, the donors had granted written declaration of informed consent. HTPCs were cultured and propagated in DMEM High Glucose (Gibco, Paisly, UK) added with 10% fetal bovine serum (Capricorn Scientific, Ebsdorfergrund, Germany), 1% penicillin/streptomycin (Biochrom, Berlin, Germany) at 37 °C and 5% (v/v) CO_2_. Purity of the cell cultures was shown previously^10^. The cells studied derived from donors, with obstructive azoospermia and normal spermatogenesis. The age of the donors ranged from 39 to 55 years. HTPCs underwent serial passaging until the typical signs of senescence appeared (growth arrest, increase in size, β-galactosidase expression, telomere shorting). All these samples were compared with the respective early passage from the same donor. The number of individual patient-derived cells for the different experiments is provided below. All experimental methods were implemented in accordance with relevant guidelines and regulations (including all biosafety and laboratory regulations).

### β-galactosidase staining

Cultured HTPCs were seeded onto coverslips. Senescence-associated β-galactosidase staining was performed using a commercial kit (Senescence β-Galactosidase Staining Kit, Cell Signaling Technology #9860, Danvers, MA, USA), according to manufacturer’s instructions. Staining was examined with a Zeiss Axiovert microscope (Zeiss GmbH, Oberkochen, Germany). HTPCs in early (P5 - P7) and advanced (P12 - P15) passages from 5 different donors were used for this experiment.

### Cell size measurement

Cell size was determined using the CASY^®^ Cell Counter on basis of variances in electrical resistance (Schärfe Systems, Reutlingen, Germany). HTPCs (from n = 7 individual donors, early (P3 – P7) and advanced (P11 – P14) passages) were trypsinized, centrifuged, resuspended in PBS and the measurement was implemented as described before^67^. For statistical analysis paired *t*-test (two-tailed) was applied.

### DNA extraction

Total DNA extraction, from HTPCs in early (P3 – P8) and advanced (P12 – P20) passages (n = 6 different donors) was carried out with Wizard^®^ SV Genomic DNA Purification System (Promega, Fitchburg, WI, USA) according to the manufacturer’s instructions.

### Relative telomere length quantification

Total DNA from HTPCs (n = 6 donors, early (P3 – P8) and advanced (P12 – P20)) was analyzed with the Relative Telomere Length Quantification qPCR Assay Kit, (Science Cell, Carlsbad, CA, USA) according to the manufacturer’s instructions. The kit contains 2 primer sets, one for the recognition and amplification of the telomere sequence and a reference primer set for data normalization. qPCR was carried out with QuantiFast^®^ SYBR Green PCR Kit (Qiagen, Hilden, Germany). A total amount of 5 ng DNA from cultured HTPCs was used in duplicates in a LightCycler^®^ 96 System (Roche Diagnostics GmbH, Penzberg, Germany) with following conditions: Initial denaturation (95 °C, 10 min) and 32 cycles of denaturation/annealing/extension (95 °C, 20 s/52 °C 20 s/72 °C 45 s). Quantification was implemented with comparative ΔΔCq method. Statistical analysis was done *via* one-sample *t*-test.

### Mitochondrial DNA copy number quantification

The mtDNA copy number was quantified by qPCR (n = 6 different donors, early (P3 – P8) and advanced (P12 – P20)). A mitochondrial and a nuclear locus were compared as described elsewhere^68^. qPCR was executed with QuantiFast^®^ SYBR Green PCR Kit (Qiagen) using 5 ng DNA and two different primer sets (for nuclear receptor coactivator three (NCOA3) and mtDNA) (Supplementary Table 7). qPCR conditions: 5 min, 95 °C preincubation, 40 cycles of amplification including denaturation at 95 °C for 10 s, annealing temperature 60 °C for 30 s and a melting step by heating from 65 °C to 95 °C, followed by a cool down to 37 °C for 30 s in a LightCycler^®^ 96 System (Roche). Comparative ΔΔCq method was used for mtDNA quantification and statistically analyzed with one-sample *t*-test.

### FIB/SEM

Cells were seeded on laser marked slides^69^. For light microscopic investigations cells were fixed in cacodylate buffer and stained with DAPI, as described by Luckner and Wanner^69^. From each passage 10 representative interphase cells were selected in phase contrast light microscopy according to their cell size and shape and documented with a CCD camera with the corresponding epifluorescence DAPI image. Cells were post-fixed/stained with either osmium tetroxide or reduced osmium-TCH-osmium (=rOTO) and ultra-thin embedded with epoxy resin as described^36,69^. After polymerization, the cells were documented again by bright field light microscopy for visualization and control of the heavy metal staining again. For high resolution SEM and FIB milling, cells were processed as described in detail by Luckner and Wanner^69^. Images were recorded with 3072 × 2048 pixel. The resulting data sets were aligned using Amira^TM^ (Thermo Fisher Scientific, Waltham, MA, USA), first automatically with the module “align slices” and corrected with the “shear” function. The quality of the alignment had to be verified manually by fine correction. Image stacks were segmented and reconstructed in Amira^TM^ (Thermo Fisher Scientific) and/or processed with a volume-rendering algorithm (*volren*) for direct visualization. 3D reconstructions/correlations were performed with Amira^TM^. Dependent on the scientific demand for 3D reconstruction pixel sizes from 7.5 µm in x/y down to 2 nm isovoxel were chosen.

### Nano LC-MS/MS

Cultured HTPCs in early (P3 – P7) and advanced (P11 – P14) passages (n = 6 different donors) were washed five times in serum-free DMEM to remove FBS and cell debris, and incubated for additional 24 h in serum-free DMEM. Cell pellets were harvested, suspended and homogenized in 8 M urea and 50 mM ammonium bicarbonate, as described previously^70^. The conditioned media (CM) were collected and centrifuged at 1,000 *g* for 3 min. The supernatant was transferred to Amicon 3 kDa centrifugal filter devices (Millipore) to desalt and concentrate the secreted proteins. The remaining concentrate solute was dried in a vacuum centrifuge. Protein concentration was determined using the Pierce 660 nm Protein Assay (Thermo Fisher Scientific)^71^. Protein samples from cells (20 µg) and their CM (10 µg) were digested in consecutive incubation steps with Lys-C (enzyme/substrate: 1:100; Wako) for 4 h at 37 °C and trypsin (enzyme/substrate: 1:50; Promega) overnight at 37 °C, as described earlier^70^. Peptide samples were analyzed by nano-LC-MS/MS on an UltiMate^TM^ 3000 RSLCnano system (Thermo Scientific) coupled to a TripleTOF^®^ 5600 + mass spectrometer (Sciex). 2.5 µg of peptides were separated at a flow rate of 200 nl/min with an analytical column (Acclaim^TM^ PepMap^TM^ RSLC C18, 75 μm × 50 cm, 2 μm, Thermo Fisher Scientific) in consecutive linear gradients: 5–25% solvent B (0.1% formic acid in acetonitrile) in 255 min and 25–50% B in 60 min. MS data were acquired in scan cycles of one survey scan (m/z 400–1250) followed by 70 data dependent CID fragmentation scans. MS raw data were processed using MaxQuant (v. 1.6.1.0). Database search parameters were set to SCIEX TOF instruments and protein identification was performed using the human Swiss-Prot subset (release 2018–10) and the MaxQuant common contaminants database at a false discovery rate of 1%. Label-free quantification (LFQ) was used as quantification strategy with a LFQ min. ratio count of 1 and the match between runs feature enabled. Data analysis and statistics was done in Perseus^72^ and R^73^. Gene Ontology (GO) and KEGG pathway annotations were retrieved from UniProt. Protein identifications in the conditioned media data were filtered for extracellular locations to define the secretome protein dataset. The presence of a signal peptide position and the keyword “secreted” in UniProt were used as indication for classical secretion. Paired *t*-tests were used to identify significantly differentially abundant proteins with an s0 value of 0.1^74^. The FDR was controlled to be < 0.05. Gene set enrichment analysis (GSEA) was done using the Reactome and KEGG databases, as well as GO categories (molecular function, biological process, cellular component). Permutation type was set to gene type, enrichment statistic was weighted and as metric a *t*-test was used, while the rest of the settings were kept at their defaults. Enrichment maps were generated in Cytoscape^75^ using the Enrichment map^76^ and clusterMaker2^77^ apps. The following settings were used for the enrichment maps: FDR q-value was 0.05 and as metric for edge generation an overlap index of 0.5 was used. REVIGO^78^ was used to summarize significant gene sets by clustering similar ontology terms.

### Immunohistochemistry

Immunohistochemical staining was performed as published previously^67^. Sections from patients with normal spermatogenesis (n = 6, age 48–50 years) were studied (Ethikkommission, Technische Universität München, Fakultät für Medizin, project number 309/14). Primary polyclonal goat anti-human DPP4 antibody (1:40, R&D Systems, Minneapolis, MN, USA) was used. For negative control purposes, the primary antiserum was omitted and replaced by non-immune serum. Hematoxilin was used to counterstain the sections. Examination of the sections was done with a Zeiss Axiovert light microscope (Zeiss GmbH).

### Reverse transcription and qPCR

RNeasy Plus Micro Kit (Qiagen) was used for total RNA isolation from HTPCs in early (P3 – P8) and advanced (P12 – P16) passages. 200 ng RNA were reverse transcribed, random 15mer primer and SuperScript^TM^ II (Invitrogen, Darmstadt, Germany). A LightCycler^®^ 96 System (Roche) and QuantiFast^®^ SYBR Green PCR Kit (Qiagen) were used. Primer are listed in Supplementary Table 7 Results were analyzed according to 2^−ΔΔCq^ method, mRNA expression was normalized to HPRT and RPL19, which served as endogenous references. Results were depicted as means ± SEM. Statistical analysis was done with one-sample *t*-test of ΔΔC_q_ values using GraphPad Prism 6.0 Software (GraphPad Software, San Diego, CA, USA). Negative controls consisted of non-reverse transcription and non-template reactions.

## Supplementary information

Supplementary Dataset 1

Supplementary Dataset 2
