# Supplementary material for: Insights into replicative senescence of human testicular peritubular cells
Source: Sci Rep. 2019 Oct 21;9:15052. doi: 10.1038/s41598-019-51380-w (PMC6803627; doi:10.1038/s41598-019-51380-w)
Supplement: Supplementary file 1 — Supplementary Dataset 1 [file 41598_2019_51380_MOESM1_ESM.docx]

**Insights into replicative senescence of human testicular peritubular cells**

Nina Schmid^1^, Florian Flenkenthaler^2^, Jan B. Stöckl^2^, Kim-Gwendolyn Dietrich^1^, Frank M. Köhn^3^, J. Ullrich Schwarzer^4^, Lars Kunz^5^, Manja Luckner^6^, Gerhard Wanner^6^, Georg J. Arnold^2^, Thomas Fröhlich^2^, Artur Mayerhofer^1*^

^1^LMU München, Biomedical Center, Anatomy III – Cell Biology, 82152 Planegg-Martinsried, Germany

^2^Laboratory for Functional Genome Analysis LAFUGA, Gene Center, LMU München, Germany

^3^Andrologicum München, 80331 München, Germany

^4^Andrologie Centrum München, 81241 München, Germany

^5^LMU München, Department Biology II, Division of Neurobiology, LMU, 82152 Planegg-Martinsried, Germany

^6^LMU München, Department Biology I, Ultrastructural Research, 82152 Planegg-Martinsried, Germany

**Corresponding author**:

Artur Mayerhofer, MD, Biomedical Center Munich (BMC), Cell Biology, Anatomy III, Ludwig-Maximilians-Universität München, Grosshaderner Strasse 9, 82152 Planegg-Martinsried, Germany

Email: [Mayerhofer@lrz.uni-muenchen.de](mailto:Mayerhofer@lrz.uni-muenchen.de); phone: +49 89 2180 75859

**Supplementary Data**

**Figure Legends**

**Supplementary Figure 1**

Selected FIB/SEM micrographs (a), volume rendering (b) and corresponding 3D-reconstruction (c, d) of a high-resolution series (4 nm isovoxel) of an advanced passage of HTPC. The numbers (a; upper right) indicate the selected micrograph from the series. A lysosome (Ly) is in contact with several strands of rough endoplasmic reticulum (ER) and a segment of a long mitochondrion (M; pink). The point of fusion of the mitochondrion with the lysosome (a; circle) is a vacuole-like structure (blue) within the mitochondrion. Volume rendering of the contact site: the vacuole within the mitochondrion is fused with the lysosome and a strand of ER is attached to the mitochondrion (b). Segmentation of the series (c) shows the interaction between lysosome, mitochondrion, vacuole, and ER both in front view and from the backside. When cut, luminal connection between the lysosome, vacuole and the mitochondrion is visible (d, arrow).

**Supplementary Figure 2**

Unsupervised hierarchical clustering indicating separation of cellular proteomes (a) and secretomes (b) from early and advanced HTPC passages. LFQ intensity values are z-score normalized and color-coded according to the expression values.

**Supplementary Figure 3**

Functional networks of gene sets disturbed in advanced compared to early HTPC passages. GSEA results from HTPC cellular proteomes (a) and secretomes (b) from early and advanced passages are visualized as networks with nodes representing significantly (FDR q-value ≤ 0.05) enriched gene sets and edges representing mutual overlap of at least 0.5. Node size scales with gene set size while edge width represents extent of overlap between gene sets. Red node color shows enrichment in advanced passages, while blue color represents enrichment in early passages. Annotation databases are indicated as GO (Gene Ontology), REACTOME or KEGG.

**Supplementary Figure 4**

ELISA-measurement of CXCL12. ELISA was performed using cell culture media of early and advanced passages of HTPCs, connected by a line; cells were incubated for 24 h.

**Supplementary Figure 5**

CCL2 ELISA-measurement. CCL2 analysis in cell culture media using cell culture media of early and advanced passages of HTPCs, connected by a line; cells were incubated for 24 h.

**Figures**

**Supplementary Figure 1**

**
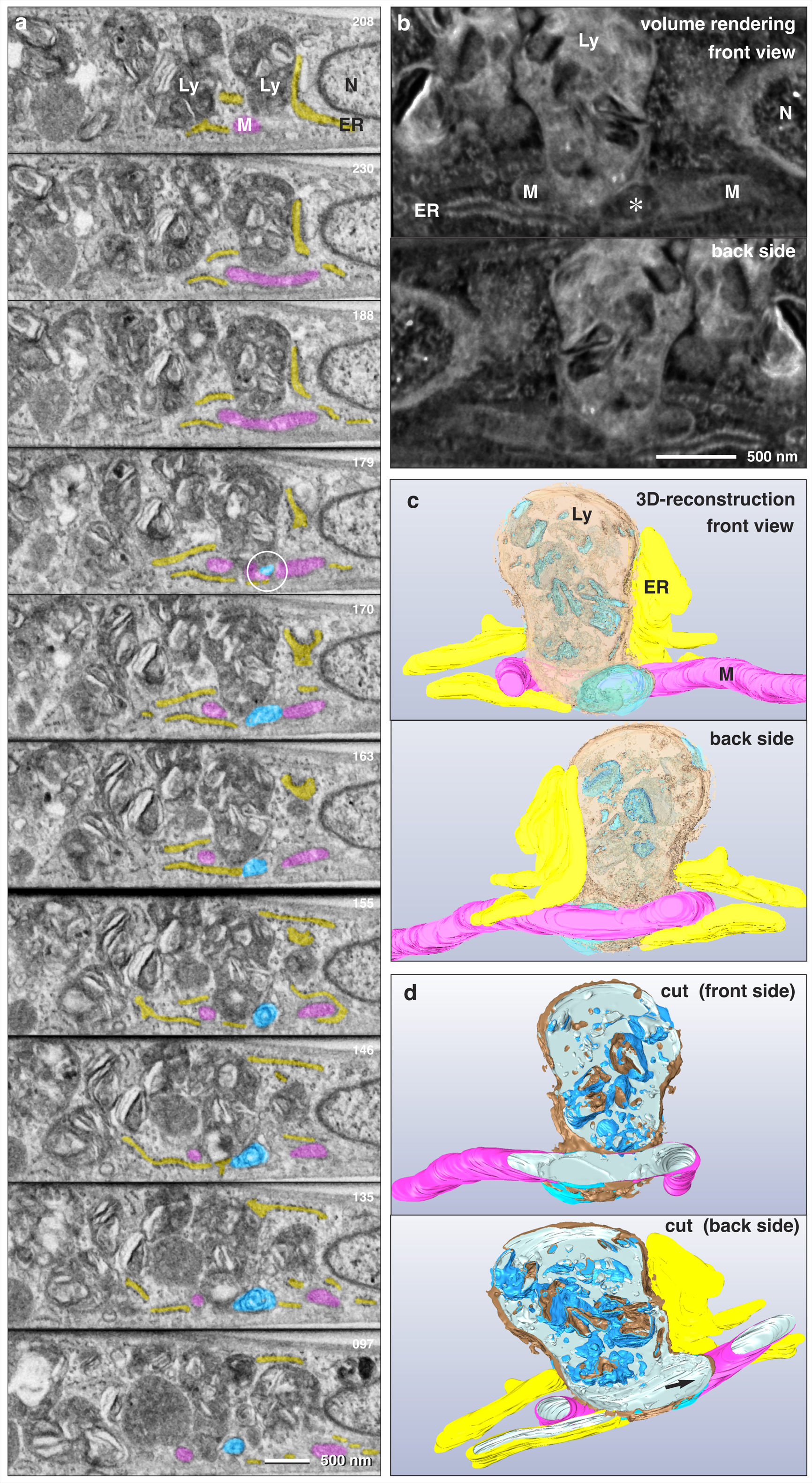
**

**Supplementary Figure 2**

**
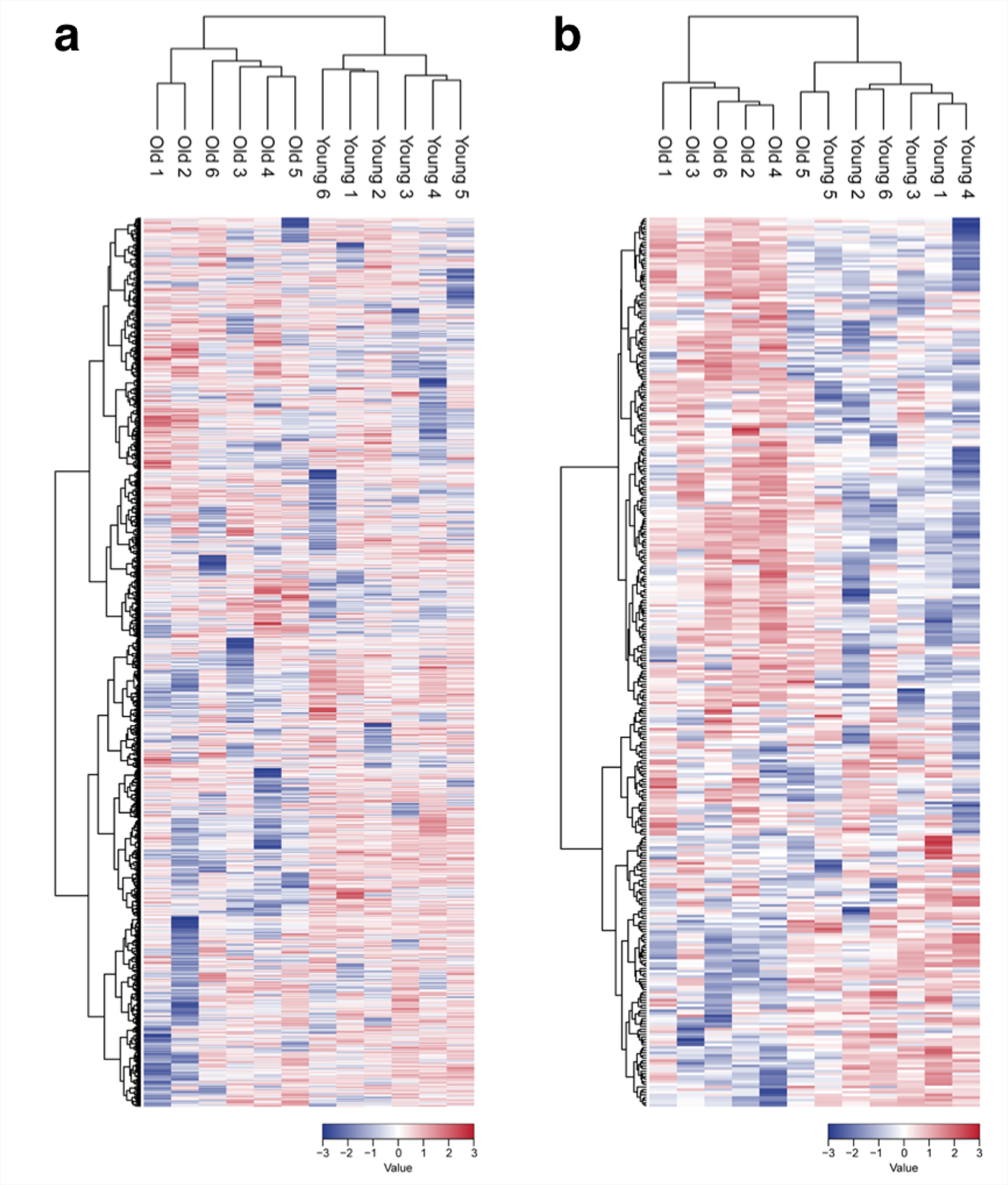
**

**Supplementary Figure 3**

**
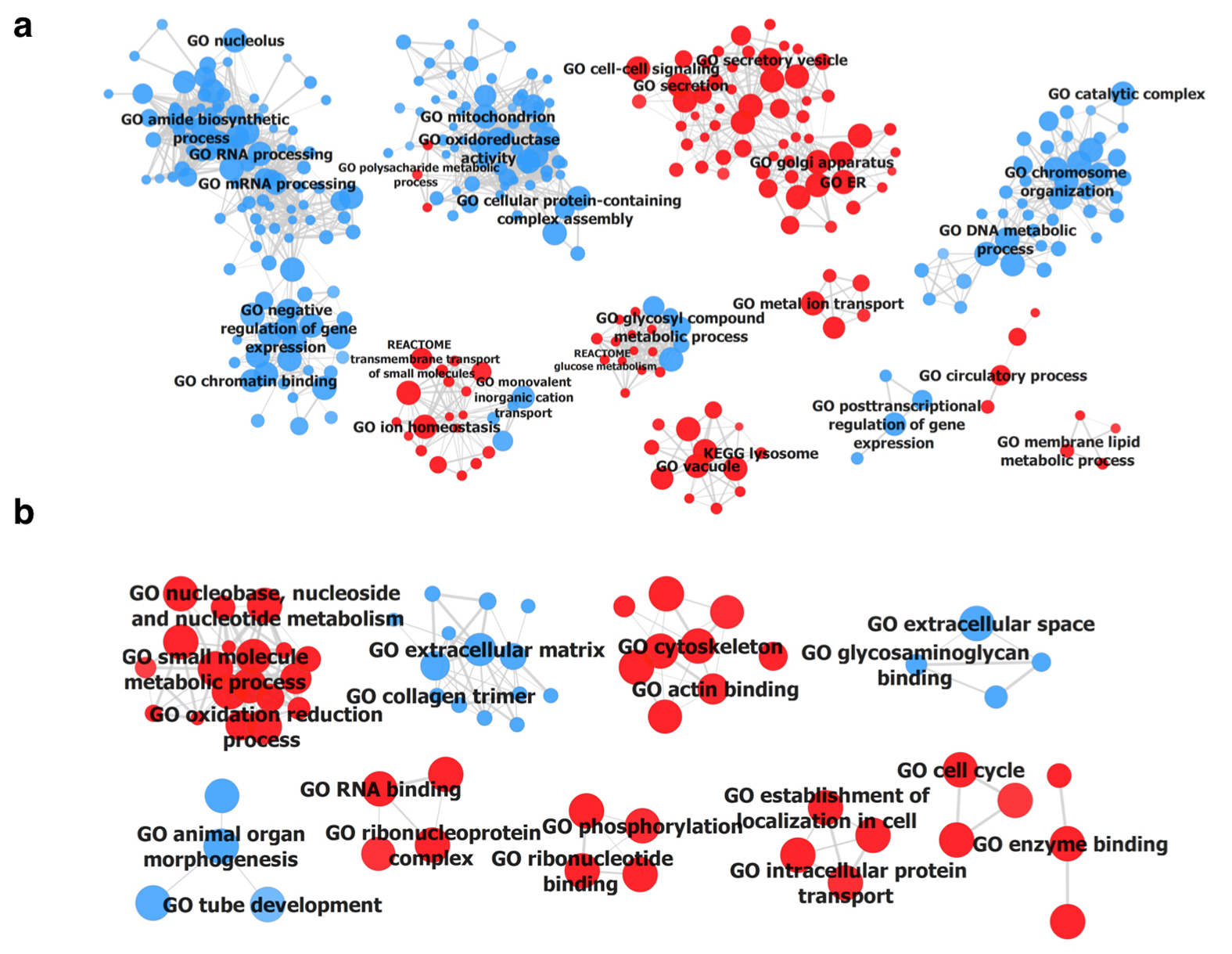
**

**Supplementary Figure 4**

**
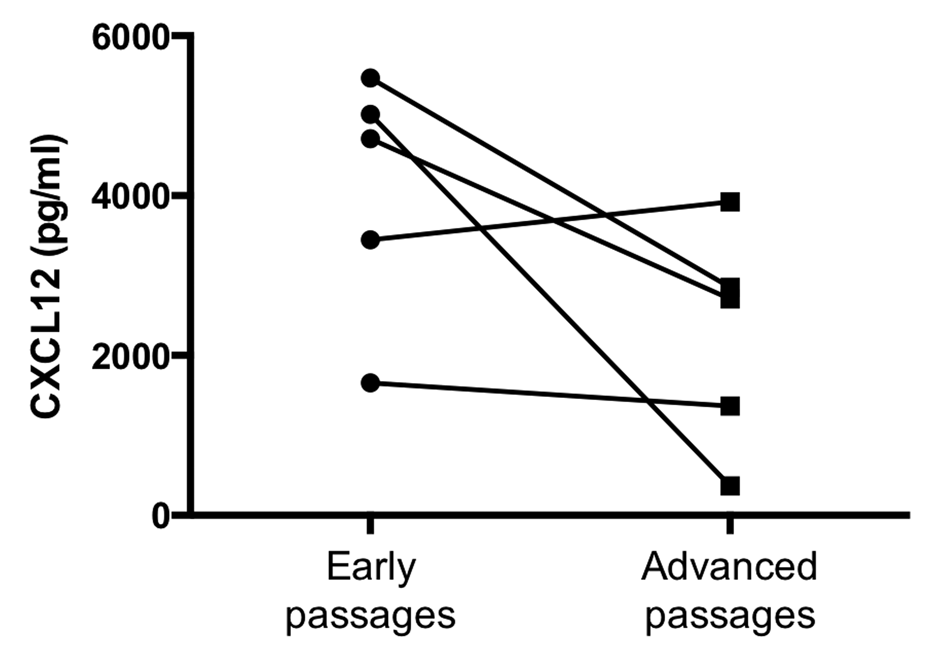
**

**Supplementary Figure 5**

**
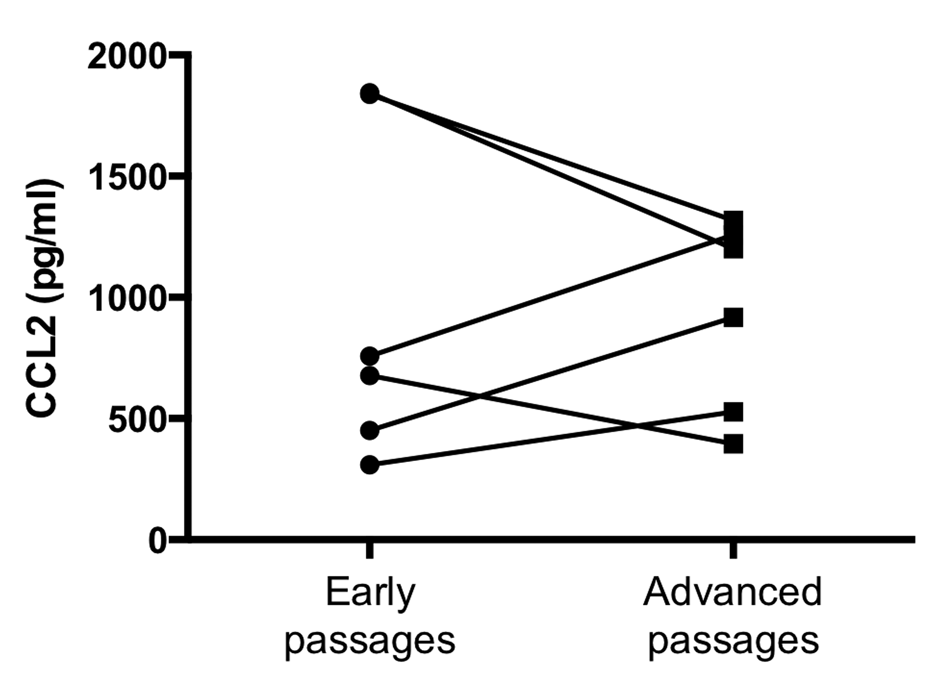
**

**Supplementary Tables**

**Supplementary Table 1.**

Proteins identified in early and advanced passages of HTPCs.

**Supplementary Table 2.**

Proteins identified in the conditioned media of early and advanced passages of HTPCs.

**Supplementary Table 3:**

Proteins significantly different in abundance (paired t-test, FDR < 0.05) between early and advanced passages of HTPCs. HTPCs are marked with a '+'. Positive log2 fold changes indicates higher abundancy in the senescent group.

**Supplementary Table 4:**

Proteins significantly different in abundance (paired t-test, FDR < 0.05) between secretomes of early and advanced passages of HTPCs are marked with a '+'. Positive log2 fold changes indicates higher abundancy in the senescent group.

**Supplementary Table 5:**

Gene set enrichment analysis from cellular proteomes from advanced vs. early passages of HTPCs. Enriched gene sets in senescent cells are listed in table A, while gene sets decreased in senescent cells are shown in table B.

**Supplementary Table 6:**

Gene set enrichment analysis from secretomes from advanced vs. early passages of HTPCs. Enriched gene sets in secretomes of senescent cells are listed in table A, while gene sets decreased in secretomes of senescent cells are shown in table B.

**Supplementary Table 7**

Oligonucleotide primer for PCR studies

| **Gene** | **Reference ID** | **Nucleotide sequence** | **Amplicon size** |
| --- | --- | --- | --- |
| *ACTA2* | NM_001613.2 | 5´-ACA ATG AGC TTC GTG TTG CC-3´  5´-GAG TCA TTT TCT CCC GGT TGG-3´ | 90 |
| *AR* | NM_001011645.3 | 5´-AGC CTC AAT GAA CTG GGA GA-3´  5´-TCC TGG AGT TGA CAT TGG TG-3´ | 175 |
| *CCL2* | NM_002982 | 5'-CAG CCA GAT GCA ATC AAT GCC-3'  5'-TGG AAT CCT GAA CCC ACT TCT-3' | 190 |
| *CNN1* | XM_005257058.4 | 5´-CGA AGA CGA AAG GAA ACA AGG T-3´  5´-GCT TGG GGT CGT AGA GGT G-3 | 186 |
| *CXCL12* | NM_000609.6 | 5´-TCA GCC TGA GCT ACA GAT GC-3  5´-CTT TAG CTT CGG GTC AAT GC-3 | 161 |
| *DPP4* | NM_001935.3 | 5´-TGG TCT CCA AAC GGC ACT TT-3´  5´-TGC CCA TGT CAC ATC ACA CA-3 | 273 |
| *GDNF* | NM_000514.3 | 5´-GCA GAC CCA TCG CCT TTG AT-3´  5´-ATC CAC ACC TTT TAG CGG AAT G-3´ | 93 |
| *HPRT* | NM_000194.2 | 5´-CCT GGC GTC GTG ATT AGT GA-3´  5´-GGC CTC CCA TCT CCT TCA TC-3´ | 163 |
| *IL6* | NM_000600.4 | 5´-AAC CTG AAC CTT CCA AAG ATG G-3´  5´-TCT GGC TTG TTC CTC ACT ACT-3´ | 159 |
| *IL8* | NM_000584.4 | 5´-TCT TGG CAG CCT TCC TGA-3´  5´-GAA TTC TCA GCC CTC TTC-3´ | 271 |
| *mtDNA* | NC_012920.1 | 5´-GCCACAGCACTTAAACACATCTCT-3´  5´-TAGGATGGGCGGGGGT-3´ | 186 |
| *NCOA3* | NC_000020.11 | 5´-CCTCTGGGCTTTTATTGCGAC-3´  5´-CGGTCATCAGAAGAACAGGTAAGT-3´ | 188 |
| *PTGS1* | NM_000962.4 | 5'-TCC ATG TTG GTG GAC TAT GG-3'  5'-GTG GTG GTC CAT GTT CCT G-3' | 96 |
| *PTGS2* | NM_000963.3 | 5´-CTT ACC CAC TTC AAG GGA-3´  5´-GCC ATA GTC AGC ATT GTA AG-3 | 132 |
| *PTX3* | NM_002852.3 | 5´-TAG TGT TTG TGG TGG GTG GA-3´  5´-TGT GAG CCC TTC CTC TGA AT-3´ | 110 |
| *RPL19* | NM_000981.3 | 5´-AGG CAC ATG GGC ATA GGT AA-3´  5´-CCA TGA GAA TCC GCT TGT TT-3´ | 199 |
| *StAR* | NM_000349 | 5´-ACG TGG ATT AAC CAG GTT CG-3´  5´-CAG CCC TCT TGG TTG CTA AG-3´ | 149 |
